# Leisure-time physical activity for middle-aged and older people with spinal cord injuries: beyond physical gains and individual responsibility

**DOI:** 10.1038/s41393-026-01263-5

**Published:** 2026-08-27

**Authors:** Laura Stendell, Camila Quel De Oliveira, Antony Jones, Peter W. Stubbs, Simon Darcy, Rachael Presdee, Robert Wynn, Anne Currie, Nathalia Costa

**Affiliations:** 1 Discipline of Physiotherapy, Graduate School of Health, Faculty of Health, University of Technology Sydney, Sydney, Australia; 2 Department of Physiotherapy, Faculty of Medicine, Dentistry and Health Sciences, University of Melbourne, Melbourne, Australia; 3 School of Health Sciences, Western Sydney University, Sydney, Australia; 4 Person with lived experience of spinal cord injury, Sydney, Australia; 5 School of Business, University of Technology Sydney, Sydney, Australia; 6 University of Queensland’s Clinical Trials Capability (ULTRA) Team, The University of Queensland, Brisbane, Australia

**Keywords:** Quality of life, Public health

## Abstract

**Study design:**

We conducted a descriptive qualitative study.

**Objective:**

To identify strategies to enhance access to and engagement in leisure-time physical activity (LTPA) for Australians aged ≥45 years of age with spinal cord injury, with the view to address the high rate of inactivity and associated health risks in this group.

**Setting:**

Community-dwelling middle-aged and older adults living in Australia.

**Methods:**

Qualitative data were gathered through focus groups and collaboratively analysed with individuals with SCI, using reflexive thematic analysis.

**Results:**

We conducted three focus groups with 19 participants ≥45 years who sustained a SCI. We identified four themes: 1) *moving to feel good, ‘normal,’ and belong* – encompassing the main motivations participants reported for engaging in LTPA; 2) *physical activity should provide opportunities for social interactions within the community and outdoors* – stressed factors that were considered important for making participation in LTPA enjoyable; 3) *accessibility and meaningful inclusion are imperative for physical activity participation* – with subthemes highlighting key factors for improving engagement in LTPA: 3.1) *welcoming communities, accessible and inclusive environments foster a sense of belonging*; 3.2) *learning from lived experience*; 4) *systemic changes are needed to enable physical activity participation*— including government funding, education, addressing societal stigma, adapting programs, providing appropriate infrastructure and equipment.

**Conclusions:**

Enhancing physical activity participation among individuals ≥45 years of age with spinal cord injury requires multifaced interventions. Education, centralised information access, collaborative approaches, accessible environments, and welcoming communities are essential for improving accessibility and promoting physical activity participation, ultimately enhancing health and well-being.

## Introduction

Individuals face several challenges after acquiring spinal cord injuries (SCI), including physical impairments, health complications, and psychological adjustments [1]. These impairments increase the risk of secondary health conditions and accelerate the onset of physiological ageing and the issues associated with it [2, 3]. Notably, the proportion of people with a SCI in their middle and older age ( ≥ 45 years) is increasing due to advancements in medical care and increased occurrence of falls, alongside the ageing of populations [4, 5]. Individuals who age with SCI are at greater risk for developing metabolic and cardiovascular diseases and a shorter lifespan than those ageing without SCI [6]. This emphasises the need to reduce health inequities experienced by people with SCI.

Middle-aged and older adults with SCI exhibit some of the lowest levels of physical activity compared to other groups of people with disability [7]. Leisure-time physical activity (LTPA) presents as an opportunity to increase daily physical activity participation as it is completed in a person’s recreation time for enjoyment, such as wheeling, participating in sports, gardening, and going to the gym, among other recreational activities [8]. Yet, in Australia, 44% of middle-aged and older adults with SCI do not engage in any weekly LTPA and 75% reported no participation moderate-to-heavy intensity LTPA [9]. Similarly, Swedish adults over 50 with a SCI at least 10 years since injury do not engage in enough LTPA to achieve health benefits [10]. Given that there are well-documented advantages of regular participation in LTPA in this population, such as decreased cardiovascular risk factors [11], enhanced mobility, and better mental well-being and quality of life [12], it is important to consider factors that create and perpetuate both physical inactivity and disability.

Individuals with SCI face many barriers to engage in LTPA [13, 14], contributing to low participation levels. Research shows that stigma in fitness centres [15], pain and fatigue during exercise [12] and psychological factors, such as fear of injury, lack of motivation and depression all impede participation [13, 14]. Social barriers, including a lack of support from family, friends, or caregivers, and low awareness of adaptive programs further restrict participation [14]. Inaccessible environments [16], lack of adaptive equipment and specialised professionals, inadequate facilities [14] and costs of programs or support workers also limit access [13]. These complex, multidimensional barriers highlight the need for targeted and collaborative interventions to support middle-aged and older adults with SCI to engage in LTPA.

Given that research with and for people with SCI is crucial to ensure their experiences are reflected in problem identification and research prioritisation [17], we argue that individuals with SCI must be involved in both the conducting of, and the design of strategies to address barriers to LTPA. Therefore, we collaborated with individuals with SCI to develop the following research question: *“What strategies can enable middle-aged and older adults to participate in accessible and enjoyable LTPA, and what forms of LTPA are most meaningful and appealing to them?”*. We then explored the perspectives of middle-aged and older adults ( ≥ 45 years) living with a SCI on what makes it accessible and enjoyable, focusing on strategies to enable their LTPA participation.

## Methods

### Study design

We conducted a qualitative descriptive study and adopted focus groups as a method to gather qualitative data. We chose a descriptive approach to collaborate with people with SCI who were not researchers, and generate practical suggestions for advocacy in the field [18]. This study draws from a constructivist paradigm, assuming that participants’ experiences of LTPA reflect multiple, socially constructed realities (relativist ontology) [19]. The knowledge presented here was co-constructed through researcher–participant interaction and interpreted through critical reflection on these multiple realities (subjective epistemology) to identify strategies to enable LTPA participation [19]. Ethical approval was obtained from the University of Technology Sydney’s human ethics board (HREC No: ETH23-8190).

### Participants and procedures

We used focus groups to explore shared experiences and generate ideas or solutions, given that they are well-suited for generating ideas for actions that may be required to address an issue [20]. Participants ( ≥ 45 years of age, living with a SCI) were recruited from across Australia from August to October 2023 through consumer organisations, investigators’ networks and a research database. Consumer organisations advertised the study through their services, online platforms, and by word of mouth. Snowballing was encouraged. Recruitment continued until we had a diverse group of participants to conduct three focus groups of 6–8 participants per group as recommended [21]. We sought to include participants varying in age (but all ≥ 45 years old), sex, neurological status (as per the International Standards for Neurological Classification of Spinal Cord Injury)[22], geographic location and socioeconomic backgrounds in order to achieve the richness and complexity needed to address the research questions [23].

Focus groups were led by an independent professional facilitator and first author (LS). The focus group included a welcome and housekeeping session, guiding principles of participation (e.g., all perspectives are welcome, everyone should have ample opportunity to voice their opinions) and confirmation that participants consented to have the session recorded. LS then outlined the aim of the focus group, defined “leisure-time physical activity”, and presented the barriers to LTPA reported in previous research as background information. After initial introductions from participants, the discussion progressed through exploratory questions about LTPA (see Table 1). All participants were reimbursed with a gift voucher (AUD$50). The focus groups were conducted via videoconferencing software, Zoom, audio-recorded and transcribed. LS checked and de-identified transcripts, assigning pseudonyms to each participant.

**Table 1:** Focus Group Questions.

Question
1 What appeals to you about participating in leisure time physical activity? Prompt: -Why would you want to engage in it? -What do you get out of it?
2 What does your ideal form of leisure-time physical activity look like? Prompt: -What are you doing? -Where are you? -Who are you with?
3 How do you think we can make it easier for people to participate in leisure time physical activity? (keeping in mind barriers listed prior)
4 We want to develop a clear plan (or intervention) to make it easier for people with a spinal cord injury to participate in and enjoy leisure time physical activity. Now we addressed some solution for the barriers, what would be a good starting point? How can we address those barriers in an intervention? Prompt: -How can we make them happen? -What would an intervention for improving leisure time physical activity look like?
5. If you could wave a magic wand and make one practical change that would make it easier for you to participate in and enjoy leisure time physical activity, what would it be?

### Researchers’ reflexivity

The research team comprised researchers (LS, NC, CQO, PS and SD), clinicians (LS and CQO), and individuals with SCI (SD, AJ, RP, RW and AC), combining lived experience (collectively spanning more than 40 years), clinical experience in neurological rehabilitation, expertise in qualitative research methods, and consumer advocacy. This multidisciplinary collaboration was instrumental in shaping the study’s design, analysis, and interpretation of findings; grounding our work in real-world experiences of navigating healthcare, rehabilitation, and community participation post-SCI. Throughout data collection and analysis, we engaged in regular reflexive discussions regarding how our professional backgrounds, personal experiences, assumptions, and interests may have influenced our interpretations. For example, LS and CQO, both physiotherapists, were particularly interested in LTPA participation in terms of intensity, enjoyment and community participation in the SCI population, while NC was drawn to the systemic and social aspects embedded in participants’ responses, such as societal stigma and socioeconomic status. AJ did not engage in LTPA and prompted us to consider why others may choose not to.

### Data analysis

We employed reflexive thematic analysis (RTA) [24], a flexible method for interpreting experiences and meanings. This approach suited our aim of exploring what makes LTPA enjoyable for middle-aged and older adults with SCI and identifying strategies to support participation. We adopted a descriptive orientation to provide a nuanced yet close-to-data account relevant to clinicians, consumers, researchers and policymakers.

We followed a six-phase iterative process [25]. Four authors (LS, NC, CQO and AJ) independently familiarised themselves with the three transcripts, noting key concepts and reflections (what stood out to them and why) before meeting to discuss these initial impressions and reflect on what experiences informed their thinking, as well as their responses to the data. LS summarised the team’s reflections and inductively coded the focus group data based on the codes and notes generated during the collaborative discussions, using NVIVO software to assist data organisation. LS then presented the resulting codes and categories to CQO, NC, and AJ, who worked together to develop theme candidates. Finally, LS drafted the results, which all authors refined. The analysis and the reporting of the results followed Braun and Clarke’s guidance [26].

## Results

Nineteen people participated in three focus groups of six to seven participants each. Participants’ age ranged from 45–72 years, with an average time since injury (standard deviation) of 15.7 (12.5) years. Participants are further described in Table 2.

**Table 2:** Participant characteristics and demographics (n = 19).

Characteristic	
Age (years), mean (SD)	61.3 (7.6)
Time Since Injury (years), mean (SD)	15.7 (12.5)
Sex, n (%)	
-Male	11 (57.9)
-Female	8 (42.1)
Level of injury, n (%)	
-Paraplegia	11 (57.9)
-Tetraplegia	8 (42.1)
Severity of injury, n (%)	
-Complete	6 (31.6)
-Incomplete	12 (63.2)
-Unsure	1 (5.3)
States of residence	
-Australian Capital Territory	1 (5.3)
-New South Wales	10 (52.6)
-Queensland	2 (10.5)
-South Australia	2 (10.5)
-Victoria	3 (15.8)
-Western Australia	1 (5.3)
Living place, n (%)	
-Metropolitan	15 (78.9)
-Rural centres	4 (21.1)

Our analysis identified four themes, which are presented below and in Table 3, illustrated by quotes from participants, who are identified under pseudonyms, with their age presented next to their name.

**Table 3:** Themes.

Theme	Quotes
1.Moving to feel good, ‘normal’ and belong	*“It makes me feel strong in mind and in the body as much as I can. If I don’t exercise (…) I get a little bit depressed,”* – *John [72]*. *“I’d reiterate what everyone said, it definitely makes a huge difference in the mood, your physical and mental mood, and just spirit.” – Greg [63]* *“I’m very much the same (…) I do a lot of gym work and it’s for my mental health. I feel really good after having pushed myself in the gym.” – Daniel [56]* *“I think the mental health of independence…I think there is a thing about just working with a body that doesn’t work very well, that’s incredibly challenging to be in. And when you can do anything well, to the best of your ability, it feels really good.” – Michelle [63]* *“I do a lot of leisure activities ranging from scuba diving to horse riding to just going to the beach and getting someone to carry me to the ocean. What I like about it is that it opens your eyes to the fact that you can still do this stuff, even though you’re in a wheelchair.” - Sarah [52]* *“In the water, I feel really free because I don’t need my sticks, I can just move a bit more freely, which I love.” –Nicole [60]* *“It’s about being as normal as you can be to life before injury”- William [52]* *“And I’ve just realised actually that my current exercise or leisure program is with mostly people who are able-bodied, similar age to me, but able-bodied. And I guess what I like about that is that it sort of makes you feel a bit normal.” – Brenda [72]*. *“I’m there as one of the girls…” - Lisa [69]*. *“I love the* [gym specialised in people with disabilities]*, it’s one of the only places I walk in and feel, ‘ah, I’m home’, you know, everybody gets it. Because most of the time, I’m the odd one out.” - Michelle [63]*
2. LTPA should provide opportunities for social interactions within the community and outdoors	*“And you’re pushing around, it’s just getting out into nature, hearing birds in the trees, everything. It just makes you feel a lot better.” – Robert [63]* *“Yeah, so like what the others said as well, it’s a sense of community. It’s a sense of being out in the sun. It’s that sense of freedom.” – Jenny [57]* *“And that’s the thing I love about cycling, like my kids do it, my wife cycles. So we cycle together a couple of times during the week… There’s a couple of well-placed coffee places that you go to and because I’ve been doing it for a while, like most people, people get to see you and go, ‘Oh, that’s an odd bike, what happened to you?’ and you sort of chat to people.” – William [52]* *“The social interaction. Get out in the sunshine and the fresh air (…) just getting out of your house, being a part of the community, not being stuck indoors. Whether it’s just going for a “walk” or going to watch some sport, going to watch the footy match or a cricket match, going and seeing a band or a musician playing somewhere. It’s just getting out and about, as opposed to being stuck indoors.” – Jim [65]* *“I used to do backstroke and laps, and the good thing about that was, it kept me fit. And, and I also had the interaction, getting out and talking to other pool goers. And I really, really enjoyed that.” – Jenny [57]* *“And that I think is a benefit that we as a collective can show the community as well, that we are up to it as much as we can. And it’s sort of, it’s like a leadership role of saying, well, this person is doing as best as he possibly can, I’d like to do that too, they say to me. And so that gives me a sense of being in the community and helping the community to progress as a whole.” – John [72]*
3. Accessibility and meaningful inclusion are imperative for LTPA participation 3.1) Welcoming communities, accessible and inclusive environments foster a sense of belonging 3.2) Learning from lived experience	***3.1:*** *“People in pools, you know, need to, you know, not just one person, but maybe a few people need to be trained in how a hoist is used. That sort of thing.”* - Brenda [72] *“Probably more assistance, and funding. Where I was going for swimming, they’ve got a ramp and they’ve got a plastic wheelchair to help you get in. But once you’re in there, you need someone to help you put some floaties on your legs and, and to be able to swim. Once I’ve got them on like, I can swim quite well.” - Daniel [56]* *“Look, it’s very hard for somebody who’s in a chair with a spinal injury to have any spontaneity or to wing anything… that can be very confronting to just sort of, you know, let’s, let’s just go to the beach and have a swim. I can’t do that! But if you can find areas and places and people where they get your limitations to a degree anyway, it just opens up a whole new world to you have fun and joy.” – Sarah [52]* **3.2:** *“It’s a bit of a tricky one because unless people have been exposed to the problems and issues with wheelchairs, they don’t fully understand until they see it.” – Scott [55]* *“It’s consultation. They don’t even consult where they’re going to put carparks, so why would they even consult us about getting into the ocean?” – Jenny [57]* *“Maybe, maybe if we put a councillor (local government representative in Australia) in a wheelchair, took them out with us, you know, they had to pretend to be us, what we can and can’t do.” – Lisa [69]* *“Sadly, one of the problems is that a lot of this stuff is planned and constructed and designed by people who are not in wheelchairs, who have never been in a wheelchair, and have no idea how hard it is to be in a wheelchair in the environments that they’re in.” – Angela [72]* *“And it’s a case of, the best disabled equipment is designed by disabled people. Whether it be snow skis, water skis, hand cycles. We’re the ones who know what we want. Whereas a lot of designers think they know what we want and design for us. But they don’t know the ins and outs and the intricacies.” – Brian [66]* *“I mean, I don’t know if you’re aware, but the beach mats (in Sydney, Australia) go halfway down the beach. So what do you do? You get halfway and then you just have to sit there and look at everybody? Sitting in the baking sun looking at everybody else swimming? … it defies logic.” - Sarah [52]* *“I don’t think we can emphasise enough the importance of feedback from us with lived experience.” – Paul [58]*
4. Systemic changes are needed to enable LTPA participation	“*you cannot compare Cairns to Sydney or Melbourne*” – Brenda [72] *“We’re able to go out and be in the community and advise people what the various needs are. And I think this it would be great if we could approach government of wherever they are, and able to make it mandatory in legislation that that if a facility is being built and whoever is building it must consult with the various forms of disability that they have to accommodate in that particular facility.” – Tom [55]* *“But if there was somewhere where we could access, okay, where are some walks around Sydney or Brisbane? Or the Blue Mountains? Where are some walks that the wheelchairs can access? Where are some beaches that wheelchairs can go to where there are mats? where are some, you know, where is Sailability [adapted sailing program in Australia] based out of?” – Lisa [69]* *“Yeah, there was a disability dance class, but it was for people with intellectual disabilities. Yeah. So you know, I don’t know how to find those things. I do yoga with someone who understands disability, that’s something that I should mention as well. For me, I do yoga.. it’s amazing. But that took me nine years to find how to do that.” - Michelle [63]* *“But the trouble is, I’m sorry. The trouble with [specialised accommodation facility that offers physical activity programs] is that its $1000 a night.” – Jenny [57]* *“My concept is to have more awareness throughout clubs (local sporting clubs) and organisations of how to integrate disabled participants into their group. At the moment, it’s always an afterthought.” – Brian [66]*

### Theme 1: Moving to feel good, ‘normal’ and belong

When discussing motivations to engage in LTPA, participants mentioned the physical benefits of LTPA (e.g., strength, reduced pain), but emphasised psychological and social gains. Most described LTPA as strengthening mental health by providing resilience, improving their sense of freedom, increasing confidence, and making them feel capable of engaging in meaningful activities. Michelle [63] noted:

*“I think the mental health of independence…I think there is a thing about just working with a body that doesn’t work very well, that’s incredibly challenging to be in. And when you can do anything well, to the best of your ability, it feels really good.*”

Here, Michelle [63] highlights how LTPA helps her adapt and create a new way of doing and being in the world despite ‘working with a body that does not work very well’. While her quote implies a preconceived idea of how bodies should move (potentially informed by socially and culturally accepted levels of normality and independence), her reference to feeling ‘good’ and doing ‘anything well’ suggests a shift from grieving a body that ‘doesn’t work well’ to focusing on what her body can do. Becoming aware of what bodies in wheelchairs can do through LTPA was commonly reported. For instance, some participants highlighted water-based activities and its liberating effect, noting that water environments felt less exclusionary and reduced reliance on mobility aids, allowing a sense of functional and existential freedom: “In the water, I feel free because I don’t need my sticks, I can just move a bit more freely, which I love” (Nicole, 60).

Overall, engaging in physical activity outside rehabilitation enabled participants to re-signify their injury by drawing attention to what they were capable of and creating different ways of living after injury, and experiencing ‘normalcy’. Feeling capable and participating in LTPA also appeared to resist ableism and assumptions about what people with SCI can or cannot do. A few felt that engaging alongside able-bodied people reduced such distinctions, while others valued being active with peers with SCI (and away from the gaze of those without), finding these settings more welcoming, supportive and less “disabling”. This highlights the diverse nature of individuals’ experiences, and that ‘sense of belonging’ can be fostered and experienced in different ways for different people.

Overall, our analysis indicates participants were primarily motivated by the emotional and mental impact of LTPA, which helped them re-imagine positive experiences with movement and sometimes resist limiting narratives of disability and normality.

### Theme 2: LTPA should provide opportunities for social interactions within the community and outdoors

Participants stressed the importance of community-based LTPA that promotes social and environmental connection, such as activities (e.g., swimming, cycling) in open spaces (e.g., beaches, national parks) that involved time with friends and family. Many appreciated the social side of LTPA, such as chatting with peers over coffee at leisure centres and expressed a desire for more outdoor opportunities that create a sense of freedom and connection with nature:

*“And you’re pushing around, it’s just getting out into nature, hearing birds in the trees, everything. It just makes you feel a lot better.” Robert* [63]

One participant (William, 52) noted that engaging in LTPA in open environments sparked curiosity from others about his story, creating conversations and fostering community dialogue that can increase disability awareness. Relatedly, they highlighted the clear need for more outdoor and community-based LTPA opportunities similar to those available to the able-bodied population.

Overall, participants emphasised that LTPA fosters a sense of community, promoting social interaction and well-being. Whether through cycling with family or sharing a coffee after an activity, LTPA served as a way to connect with others, fostering belonging and freedom. One participant (John, 72) alluded to the idea that people with disabilities can serve as leaders by demonstrating their capabilities and challenging societal assumptions about individuals with SCI. In summary, participants highlighted the importance of designing spaces and LTPA programs that support social connection and integration in community and outdoor environments, helping raise awareness and inspire others.

### Theme 3: Accessibility and meaningful inclusion are imperative for LTPA participation

Our analysis identified key factors to promote inclusivity within physical activities, described under the subthemes below:

#### 3.1) Subtheme 1. Welcoming communities, accessible and inclusive environments foster a sense of belonging

Participants discussed a welcoming environment and atmosphere as vital for promoting LTPA engagement among people with SCI over 45. Within this context, disability awareness education in both the general community and LTPA venues was viewed as essential to alleviate anxiety and enhance inclusivity. Local council leisure centres were discussed as good places to engage in LTPA because they support social interaction, but participants emphasised that these centres must be accessible and that staff should be trained in inclusive practices. They suggested that existing community programs could be strengthened through disability awareness education and training. Likewise, participants discussed the need for additional assistance within current LTPA facilities. While infrastructure such as ramps and pool wheelchairs may support physical access, further help such as support staff was often required to enable full participation. This highlighted barriers that extend beyond physical accessibility:

*“People in pools need to, not just one person, but maybe a few people need to be trained in how a hoist is used.” Brenda [72]*

Some highlighted that understanding and accommodating communities and environments as key to support spontaneous, enjoyable and fulfilling LTPA. Participants expressed desire for support systems and communities that recognise and accommodate diverse needs, ultimately enriching the lives of individuals with disabilities and enhancing their sense of belonging and participation in society. Overall, this sub-theme stresses the importance of not only providing accessible infrastructure but also ensuring that staff involved in LTPA are knowledgeable and equipped to assist individuals with diverse needs.

#### 3.2) Subtheme 2. Learning from lived experience

*“Maybe, maybe if we put a councillor (local government representative in Australia) in a wheelchair, took them out with us, they had to pretend to be us, what we can and can’t do.” Lisa, [69]*

Participants highlighted the importance of meaningfully including the perspectives of people with SCI to co-design LTPA interventions that meet their needs. They advocated for early consultation with consumer organisations and wheelchair users to shape accessible activities, outdoor environments, and community spaces. Some expressed frustration at policymakers’ reluctance to consult people with disabilities about infrastructure challenges.

Participants also raised the need for advocacy from consumer organisations for more appropriate and accessible outdoor and community opportunities, shifting responsibility for accessibility away from individuals. They noted that many facilities overlook wheelchair users’ needs, underscoring the importance of incorporating lived experience in designing LTPA settings. Several participants noted that consultation is frequently an afterthought, with a disconnect between designers and users. Frustrations about incomplete or poorly executed accessibility solutions, such as beach mats that do not reach the ocean, highlighted the need for meaningful involvement of those with lived experience.

### Theme 4: Systemic changes are needed to enable LTPA participation

Participants highlighted several changes needed to improve LTPA for middle-aged and older adults with SCI: broader access to government funding for LTPA programs, centralised information about LTPA opportunities across cities and states, and education strategies to address stigma around disability and promote the benefits of LTPA. They noted existing programs should be adapted to be more inclusive, directing funding towards appropriate infrastructure, trained staff, and accessibility equipment in both community and outdoor settings. Participants often reported difficulty in finding LTPA opportunities that suited their needs, noting the time it can take to locate options with knowledgeable instructors, as well as high costs that can make these unviable for many:

*“But the trouble is, I’m sorry. The trouble with [specialised accommodation facility that offers physical activity programs] is that its $1000 a night.” Jenny [57]*

Some participants proposed mandatory consultation with disability representatives when new facilities are built to ensure accessibility and underscored the importance of investment in infrastructure such as ramps, elevators, and adaptive equipment. Yet they stressed that while such strategies can remove physical barriers, they do not necessarily support integration, with relevance for multimodal strategies that include cost subsidies, greater access to information about LTPA and education to both reduce stigma and promote LTPA.

## Discussion

Our findings emphasised the benefits of LTPA for enhancing well-being and social connectedness of individuals ageing with SCI. Participants identified strategies to improve LTPA participation, including calls for inclusive spaces and systemic changes such as funding, education to reduce stigma, better access to LTPA information resources/opportunities, program adaptations and consultation with people with SCI.

Our study identified social interactions and outdoor LTPA as key motivators for engaging in LTPA, aligning with previous research that highlights the role of environmental opportunities and social support in reducing perceived barriers to LTPA [27]. Enjoyment and “having fun” plays a central role in making LTPA appealing and sustainable, and is associated with participation in moderate-to-heavy LTPA in adults with SCI [28]. Together, creating fun, socially connected, and age-inclusive environments is critical to enabling participation among middle-aged and older adults with SCI, and may transform the way and the frequency they engage in LTPA.

Previous research emphasised the need for a comprehensive approach that considers individual and socio-environmental factors to support independence, including accessibility, tailored support, and inclusive communities for healthy ageing in individuals with SCI [29]. Our study supports these findings and highlights the need to advocate for more inclusive environments and co-designed programs/spaces to better meet their needs. Relatedly, it is important to integrate individuals with SCI into community sporting clubs, both “able-bodied” or otherwise [30]. Initiatives like “*Access for All Abilities*” in Victoria, Australia, promote inclusivity and tailored support, while community leaders and local governments can endorse LTPA opportunities. A collaborative approach to raising awareness and integrating individuals with SCI into existing sports clubs can create a more inclusive community, likely improving quality of life for people with SCI and other physical disabilities. Some of the solutions for improving LTPA identified in our study, including the need for accessible infrastructure and trained staff at LTPA facilities, have been previously explored. For example, a previous Australian study on beach accessibility for people with disabilities showed that innovative inclusive designs were essential to counter the social and environmental challenges [31]. Improving infrastructure not only benefits individuals with SCI, but also improves accessibility for the broader community (e.g. those with prams, the elderly, those with reduced mobility). It has been argued that governments and policymakers must implement consumer-driven strategies to optimise LTPA participation [32]. Our findings extend this work by demonstrating that co-design with people with lived experience could strengthen LTPA participation, likely reducing the impact of inactivity and feelings of inadequacy and isolation. Likewise, a supportive community can play a key role in creating positive LTPA experiences, such as when surfing or going to the beach [31]. Drawing from our findings and from existing literature, disability and inclusion awareness training should be implemented for staff at facilities offering LTPA opportunities. Training could educate staff on how to cater for individuals with significant mobility restrictions, reduce stigma, raise awareness about appropriate language, and assist with physical barriers (e.g., using a hoist to enter a pool). This training could reduce psychological stress and fear, providing safer spaces and promoting greater participation. For older adults, such support may also reduce anxiety about injury, safety and physical assistance needs. Inclusive marketing campaigns targeting LTPA can also normalise participation and address societal stigma. Diversifying the workforce and incorporating disability epistemology into the healthcare culture are also likely to be crucial in order to do so [33].

### Strengths and limitations

A major strength of this study is the involvement of people with SCI as co-authors - they helped to develop the research question, the discussion guide used in focus groups and the data analysis – having individuals with SCI in the research team pushed us to look at the ‘problem of physical inactivity’ through different lenses. We used the data presented here to write a policy brief and advocate for action in this space [18]. Our study participants had different levels and severities of SCI, as well as a range of different experiences, resulting in a range of solutions that may better address real-world needs. Yet, the study’s focus on Australian, predominantly metropolitan populations limits the transferability of findings to other countries, socioeconomic and political contexts. Additionally, it is important to acknowledge that individuals who engage in LTPA may have been more inclined to participate in our study. That said, recruitment strategies explicitly sought both exercisers and non-exercisers, and discussions reflected a range of perspectives on LTPA participation.

Drawing on Tracy’s Big-Tent Criteria for excellent qualitative research [23], this study demonstrated several indicators of qualitative quality. First, it examined a worthy topic identified as a priority by people ageing with SCI from a Spinal Cord Injuries Australia (a non-for-profit consumer organisation) member survey, a population that remains underrepresented in research and practice despite experiencing significant health and participation inequities. The study demonstrates rich rigor through the recruitment of participants from across Australia, the generation of rich data through multiple focus groups, and collaborative analysis involving researchers, clinicians and people with lived experience. Sincerity was supported through reflexivity [24], with the research team utilising their clinical, research, advocacy, and lived-experience perspectives to shape the research process and interpretation of findings. Credibility was enhanced using detailed participant quotations and collaborative interpretation of the data. The findings offer resonance and potential transferability by identifying barriers and facilitators to LTPA that may be relevant to other populations experiencing disability-related participation challenges. The study also provides a significant contribution by generating practical, consumer-informed recommendations and demonstrating the value of participatory research approaches in promoting LTPA participation. Ethical practice was supported through meaningful involvement of people with SCI throughout the research process alongside formal ethical approval procedures. Finally, the study demonstrated meaningful coherence through alignment between its constructivist qualitative design, reflexive thematic analysis approach, and the conclusions drawn from the data.

### Future recommendations for systemic change

Equitable access to LTPA can be supported by expanding funding opportunities (e.g. through disability insurance schemes) to improve access to support workers and to facilities catering for people with disabilities. Advocacy bodies and consumer organisations can help build the case for policy and funding changes, as well as disseminate comprehensive LTPA information. Developing centralised, easy-to-navigate physical activity resources is particularly important for older adults, who may benefit from clear guidance on where and how to access existing LTPA opportunities locally.

## Conclusion

This study highlighted the need for multifaceted interventions aimed at enabling engagement in LTPA among middle-aged and older adults with SCI. Key strategies include education initiatives to address stigma and foster welcoming communities, collaborative approaches, creating accessible environments, improving funding, adapting existing programs and sporting clubs and increasing availability of support staff in LTPA facilities. These measures are important for promoting LTPA participation amongst individuals ageing with SCI, reducing inequities and improving their health and well-being.

## Data Availability

The datasets generated during and/or analysed during the current study are available from the corresponding author on reasonable request.
